# Microstructural Tissue Changes in a Rat Model of Mild Traumatic Brain Injury

**DOI:** 10.3389/fnins.2021.746214

**Published:** 2021-11-26

**Authors:** Karthik Chary, Omar Narvaez, Raimo A. Salo, Isabel San Martín Molina, Jussi Tohka, Manisha Aggarwal, Olli Gröhn, Alejandra Sierra

**Affiliations:** ^1^A. I. Virtanen Institute for Molecular Sciences, University of Eastern Finland, Kuopio, Finland; ^2^Russell H. Morgan Department of Radiology and Radiological Science, The Johns Hopkins University School of Medicine, Baltimore, MD, United States

**Keywords:** traumatic brain injury, fixel-based analysis, diffusion tensor imaging, axonal damage, gliosis, histology, neurite orientation dispersion and density imaging

## Abstract

Our study investigates the potential of diffusion MRI (dMRI), including diffusion tensor imaging (DTI), fixel-based analysis (FBA) and neurite orientation dispersion and density imaging (NODDI), to detect microstructural tissue abnormalities in rats after mild traumatic brain injury (mTBI). The brains of sham-operated and mTBI rats 35 days after lateral fluid percussion injury were imaged *ex vivo* in a 11.7-T scanner. Voxel-based analyses of DTI-, fixel- and NODDI-based metrics detected extensive tissue changes in directly affected brain areas close to the primary injury, and more importantly, also in distal areas connected to primary injury and indirectly affected by the secondary injury mechanisms. Histology revealed ongoing axonal abnormalities and inflammation, 35 days after the injury, in the brain areas highlighted in the group analyses. Fractional anisotropy (FA), fiber density (FD) and fiber density and fiber bundle cross-section (FDC) showed similar pattern of significant areas throughout the brain; however, FA showed more significant voxels in gray matter areas, while FD and FDC in white matter areas, and orientation dispersion index (ODI) in areas most damage based on histology. Region-of-interest (ROI)-based analyses on dMRI maps and histology in selected brain regions revealed that the changes in MRI parameters could be attributed to both alterations in myelinated fiber bundles and increased cellularity. This study demonstrates that the combination of dMRI methods can provide a more complete insight into the microstructural alterations in white and gray matter after mTBI, which may aid diagnosis and prognosis following a mild brain injury.

## Introduction

Magnetic resonance imaging (MRI) and computed tomography (CT) are routinely used to assess tissue damage in patients after traumatic brain injury (TBI; Duhaime et al., 2010). While these imaging methodologies can assess tissue damage after moderate and severe injury, mild TBI (mTBI) remains a challenge by not providing clear radiological evidence of brain injury (Mittl et al., 1994; Iverson et al., 2000; Scheid et al., 2003; Hughes et al., 2004). Clinically, mTBI is defined by initial brief, decreased, or no loss of consciousness, disorientation, or amnesia, which tend to disappear within minutes or hours after injury (Mechtler et al., 2014; Pervez et al., 2018). However, many patients complain about persisting symptoms for days or even months after the injury, such as headache, dizziness, concentration/memory problems, or other long-term complications, such as sleeping disorders, emotional distress, depression, or anxiety (Katz et al., 2015; Ling et al., 2015; Cole and Bailie, 2016; van der Naalt et al., 2017). These short- and long term-consequences after a mild injury indicate that there are still ongoing processes in the brain, which are not detected by the clinically available imaging methods.

Diffusion MRI (dMRI) detects the displacement of water molecules, which reflects tissue microstructure. Therefore, changes in the tissue microenvironment can provide non-invasively detectable information associated to pathological processes ongoing in the tissue. In particular, diffusion tensor imaging (DTI; Basser et al., 1994) has demonstrated a good sensitivity to reveal microstructure-associated changes of pathological features of mTBI both in patients (Inglese et al., 2005; Yuh et al., 2014; Asken et al., 2018; Wallace et al., 2018; Yin et al., 2019) and animal models of TBI (Bennett et al., 2012; Hylin et al., 2013; Stemper et al., 2015; Herrera et al., 2017; Hutchinson et al., 2018). Despite being widely used, the single tensor model assumes one water pool with Gaussian distribution, which oversimplifies the highly complex architecture of the tissue within a voxel. For example, it has been studied that white matter voxels may contain up to 90% of crossing fibers (Jeurissen et al., 2013). Therefore, differences detected in a region-of-interest (ROI) or voxel-based analyses are confounded by partial volume effects making interpretation of DTI outcomes a challenge in the presence of multiple fiber bundles or in the gray matter (Jones et al., 2013). Recently, the introduction of more advanced techniques, such as high-angular-resolution diffusion imaging (HARDI) acquisitions (Tuch et al., 2002) in combination with higher-order diffusion modeling tools, such as constrained spherical deconvolution (CSD; Tournier et al., 2004, 2007), Q-ball (Tuch, 2004) or persistent angular structure-MRI (Jansons and Alexander, 2003), offer new windows to estimate the more complex microstructural environment of the brain tissue. Based on CSD, Raffelt et al. (2012) introduced a novel statistical analyses method for HARDI data known as fixel-based analysis (FBA), which enables quantification of individual fiber bundle populations within a voxel. More specifically, the FBA framework provides estimation of microstructural changes in apparent fiber density and macroscopic changes in fiber bundle cross-section (Raffelt et al., 2012, 2017). On the other hand, multi-compartment models, such as neurite orientation dispersion and density imaging (NODDI; Zhang et al., 2012), can extract information of volume fractions of isotropic, hindered, and restricted compartments and identify microstructural features associated with pathological process occurring in the brain. Both FBA framework and NODDI has been already used to assess tissue alterations after TBI in both animals and humans (Wright et al., 2017; Churchill et al., 2019; Verhelst et al., 2019; Gazdzinski et al., 2020; Palacios et al., 2020; Wallace et al., 2020; McCunn et al., 2021; Muller et al., 2021; Oehr et al., 2021), however, there are few studies including corroboration of the tissue changes after brain injury with histology.

The aim of our study was to investigate microstructural tissue changes throughout the brain in an experimental mTBI rat model using DTI-, fixel- and NODDI based analyses. We performed voxel-based analysis (VBA) comparing *ex vivo* sham-operated and mTBI brains of the DTI-based metrics: fractional anisotropy (FA), and axial (AD), radial (RD) and mean (MD) diffusivities; fixel-based metrics: fiber density (FD), fiber bundle cross-section (FC), and fiber density and fiber bundle cross-section (FDC); and NODDI metrics: orientation dispersion index (ODI), free water fraction (FWF), and neurite density index (NDI). Histologically, we confirmed axonal alterations and increased cell density associated to gliosis in highlighted brain areas in both analyses by using myelin and Nissl stainings, respectively. Additionally, we performed an ROI analysis on MRI and histology in the same animals, and correlated values from individual MRI maps with ones obtained from histological sections stained for myelin and Nissl. Altogether, this study explores the potential of established and advanced diffusion MRI techniques with histological validation, and more importantly, increases our understanding of tissue microstructural changes in the brain after mild injury.

## Materials and Methods

### Animal Model

Experimental TBI was induced in male Sprague-Dawley rats (*n* = 8; 10 weeks old, 300–350 g, Harlan Netherlands B.V., Horst, Netherlands) by lateral fluid percussion (LFP) injury. Rats were anesthetized with a single i.p. injection (6 ml/kg) of a mixture containing sodium pentobarbital (Mebunat, Orion Pharma, Finland; 60 mg/kg), magnesium sulfate (127.2 mg/kg), propylene glycol (39.5%), and absolute ethanol (10%). Then, a craniectomy (∅ = 5 mm) was performed between bregma and lambda on the left skull convexity (anterior edge 2.0 mm posterior to the bregma; lateral edge adjacent to the left lateral ridge). A fluid percussion device (AmScien Instruments, Richmond, VA, United States) was then used to induce an LFP injury to the exposed dura using a transient fluid pulse (21–23 ms) to induce a mild injury (0.89 ± 0.21 atm). After the injury, we checked that the dura was intact. Sham-operated rats (*n* = 6) were subjected to same operational procedures except for the impact.

Following the operation, the animals were transferred to the animal facility and housed in individual cages maintained under a 12 h light/12 h dark cycle (lights on 07:00 a.m., temperature 22 ± 1°C, humidity 50–60%) with free access to food and water. All animal procedures were carried out under licenses that have been approved by the Animal Ethics Committee of the Provincial Government of Southern Finland and in accordance with the guidelines of the European Community Council Directives 86/609/EREC.

### Tissue Preparation

Thirty-five days after the operation, all the rats (*n* = 14) were deeply anesthetized under 5% isoflurane in 30%/70% O_2_/N_2_ gas mixture, and transcardially perfused with saline for 2 min (30 ml/min) followed by 4% paraformaldehyde in 0.1 M phosphate buffer, pH 7.4 (30 ml/min) for 25 min. After perfusion, the brains were removed from the skull and post-fixed in a solution of 4% paraformaldehyde until imaging. Before MRI, the brains were transferred to a solution of 0.1 M phosphate-buffered saline (PBS) containing 1 mM gadopentetate dimeglumine (Magnevist, Berlex Imaging, Wayne, NJ, United States) for at least 72 h. The brains were then placed tightly inside a polyethylene tube filled with perfluoro polyether (Fomblin, Solvay Inc., Princeton, NJ, United States) to prevent tissue drying and to effectively suppress the background signal.

### *Ex vivo* Magnetic Resonance Imaging Acquisition

The brains were scanned on an 11.7 T NMR spectrometer (Bruker BioSpin, Billerica, MA, United States), with a Micro2.5 gradient system (maximum gradient strength = 1,000 mT/m). A 20-mm-diameter birdcage volume coil was used for radiofrequency transmission and signal reception. Diffusion data were acquired using a 3D diffusion-weighted gradient- and spin-echo (DW-GRASE) sequence (Aggarwal et al., 2010) with TR/TE = 800/33 ms, rare-factor/EPI factor = 4/3, bandwidth = 100 kHz, number of averages = 2, FOV = 22.8 mm × 16.8 mm × 11.7 mm, matrix size (read × phase × phase 2) = 152 × 112 × 78, acquired spatial resolution = 150 μm^3^ isotropic (zero-filling interpolation to 0.075 mm^3^ isotropic), number of uniformly distributed diffusion directions = 30 for each *b*-value of 3,000 and 6,000 s/mm^2^, number of minimally diffusion-weighted images = 3, diffusion gradient duration (δ)/separation (Δ) = 5/12 ms, total acquisition time ∼21 h.

### Image Pre-processing

k-space data were processed using in-house code in IDL (ITT Visual Information Solutions, Boulder, CO, United States) to reconstruct the diffusion weighted images. Preprocessing of the reconstructed data consisted of image denoising based on random matrix theory (Cordero-Grande et al., 2019) and Gibbs ringing removal using the method of local subvoxel-shifts (Kellner et al., 2016), both tools included in the MRtrix3 software (Tournier et al., 2019). Finally, bias field correction was applied to remove spatial intensity inhomogeneities (Tustison et al., 2010) followed by motion and eddy current correction using Advanced Normalization Tools (ANTs) software (Avants et al., 2014).

### Fixel-Based, Tensor-Based and Neurite Orientation Dispersion and Density Imaging Analyses

To perform FBA (Raffelt et al., 2012), tissue specific response functions were estimated for white matter (WM), gray matter (GM), and PBS in an unsupervised manner for each sham-operated animal (Dhollander and Connelly, 2016), and combined to create group averaged response functions. Whole brain masks were obtained for each image. The fiber orientation distributions (FODs) with a spherical harmonic degree (lmax = 6) were then estimated from the group averaged response functions using multi-shell, multi-tissue constrained spherical deconvolution (MSMT-CSD; Jeurissen et al., 2014). The resulting FODs were corrected for intensity inhomogeneities using multi-tissue-informed intensity normalization.

#### Unbiased Population Template

The FOD maps from all the brains, including sham-operated and mTBI, were then co-registered in two steps; first with rigid and affine registration followed by a non-linear registration to optimize a group-average template. The linear and non-linear warps generated during co-registration were subsequently used to warp (without orientation) the intensity normalized FODs from each rat to the template.

#### Fixel-Based Metrics: Fiber Density, Fiber Bundle Cross-Section, Fiber Density and Fiber Bundle Cross-Section

To ensure that further analysis is performed on fixels that contain data from all the rat brains, all the individual masks were warped to the template, and the intersection of the masks were computed to obtain a population template mask. Then, a white matter template fixel mask was created by segmenting the peak FOD amplitudes of each fixel in the FOD based template, at a threshold of 0.06. The selected threshold for the fixel mask was chosen to include fixels in crossing fiber areas that are genuinely in WM and areas with a mixture of WM and GM, without the inclusion of any spurious fixels. Each FOD lobe from the warped individual rat brain FODs were segmented into corresponding fixels by taking the integral of the FOD lobe (at the threshold defined previously) to obtain the FD per fixel and reoriented into the template image (Raffelt et al., 2012). The fixel spatial correspondence was achieved by taking each reoriented fixel in the individual rat brain FODs and assigning it to the corresponding fixel in the FOD template (Raffelt et al., 2017). The FC metric was computed from the deformation fields obtained during co-registration (from the individual to template). Finally, FDC metric was computed by modulating the FD with FC computed previously.

#### Tensor-Based and Multicompartment Model Neurite Orientation Dispersion and Density Imaging Analyses

We calculated tensor-based metrics using both *b*-values (i.e., 3,000 and 6,000 s/mm^2^) and the iteratively weighted least-squares (IWLS) method to improve accuracy in the parameter estimations (Basser et al., 1994; Veraart et al., 2013). The tensor-based metrics included were fractional anisotropy (FA), axial diffusivity (AD), radial diffusivity (RD), and mean diffusivity (MD). Multicompartment model NODDI was computed and fitted using the NODDI toolbox (UCL, United Kingdom) for Matlab^1^. The derived NODDI indices included orientation dispersion index (ODI), free water fraction (FWF), and neurite density index (NDI). For the voxel-wise analysis, the tensor- and NODDI-based metrics were then warped to the FOD-based template using the non-linear transformations obtained during FBA analysis.

### Statistical Analysis

Statistical analysis for fixel-based metrics were performed by connectivity-based fixel enhancement (CFE) which uses whole brain tractography-derived connectivity information to infer the amount of cluster-like local spatial support for each corresponding voxel (Raffelt et al., 2015). For this purpose, whole-brain tractography was performed on the study-specific FOD template within the template mask intersection using the iFOD2 algorithm by generating 20 million streamlines, angle = 22.5°, minimum length = 0.3 mm, maximum length = 22.5 mm, cutoff = 0.06, power = 1. In order to minimize errors between tractography-derived streamline densities and spherical deconvolution-derived fiber densities, the resulting tractogram was reduced to 2 million streamlines using spherical-deconvolution-informed filtering of tractograms (SIFT; Smith et al., 2013). Connectivity-based fixel enhancement was performed for the group analysis FD, FC, and FDC metrics comparing sham-operated versus mTBI rats applying a spatial Gaussian filter of 0.3 mm × 0.3 mm × 0.3 mm and using general linear model with non-parametric permutation testing (5,000 permutations) using the default MRtrix3 *fixelcfestats* tool parameters (*C* = 0.5, *E* = 2, *H* = 3) (Raffelt et al., 2015). For voxel-based metrics, a spatial Gaussian filter of 0.3 mm × 0.3 mm × 0.3 mm was applied to the data and the statistical analysis was performed with non-parametric permutation testing (5,000 permutations) using threshold-free cluster enhancement (TFCE) with the default FSL *randomize* tool parameters (*C* = 6, *E* = 0.5, *H* = 2) (Smith and Nichols, 2009). Both the DTI- and fixel-based voxel-wise statistical analyses were fully corrected for family-wise error (FWE). A *p*-value of less than 0.05 was considered statistically significant.

We pre-selected the regions of interest based on histology in the same animals as in MRI. We selected the corpus callosum and external capsule at −1.80 and −3.50 mm from bregma, and internal capsule and ventrobasal complex at −3.50 mm from bregma, both ipsi- and contralaterally. A single set of ROIs were manually drawn in the template. The selection of ROIs was based on our previous study (San Martín Molina et al., 2020). Then, the ROIs were transformed to the subject space using the inverse matrix transformation for each brain. The ROI-based analysis for histology comparison was performed for tensor-based (FA, RD, AD, and MD) and CSD-based metrics (FD, dispersion, and peak), as well as NODDI parameters (ODI, FWF, and NDI). The ROI-based analysis of FC and FDC were also included but note that these metrics requires the non-linear transformations used to generate the template, hence, the corresponded ROIs were calculated in template space.

### Histological Procedures and Analysis

The histological procedures and ROI analysis were presented in our previous study (San Martín Molina et al., 2020). In brief, after *ex vivo* imaging, the brains were washed in 0.9% NaCl for at least for 2 h at 4°C. After this, they were placed in a cryoprotective solution containing 20% glycerol in 0.02 M potassium phosphate-buffered saline (pH = 7.4) for 36 h. Then, the brains were blocked, frozen in dry ice, and preserved at −70°C until sectioning. The brains were sectioned in the coronal plane (30 μm, 1-in-5 series) using a sliding microtome. Sections from the first series were stored in 10% formalin while the remaining series were stored in a cryoprotectant tissue-collecting solution (30% ethylene glycol, 25% glycerol in 0.05 M sodium phosphate buffer) at −20°C until further processing. The first series of sections was stained with Nissl (thionin) and the second series with a gold chloride solution for myelin (Laitinen et al., 2010).

For the quantitative analysis, we selected brain areas that showed microstructural changes in the group analysis and were of interest in the TBI pathology (Laitinen et al., 2015; San Martín Molina et al., 2020; Chary et al., 2021). These brain areas were the corpus callosum and external capsule at −1.80 and −3.50 mm from bregma, internal capsule and ventrobasal complex at −3.50 mm from bregma. All the analyses were performed on high-resolution photomicrographs of the sections acquired at a resolution of 0.013 μm^2^/pixel using a light microscope (Zeiss Axio Imager 2, White Plains, NY, United States) equipped with a digital camera (Zeiss Axiocam color 506). We performed structure tensor (ST) and derived the anisotropy index (AI; Budde et al., 2011), which correlates strongly with alternative dispersion measures, from myelin-stained sections, and cell density (CD) from Nissl-stained sections using an in-house Matlab code for automated cell counting analysis (San Martín Molina et al., 2020).

### Region-of-Interest Statistical Analyses

All data were analyzed using GraphPad Prism software (version 5.03 for Windows, La Jolla, CA, United States). Differences between sham-operated and mTBI rats were assessed using the unpaired two-sample *t*-test, and differences between ipsi- and contralateral brain areas within the same brain using the paired *t*-test. Pearson’s correlation was used to correlate dMRI and histological metrics. The Benjamini-Hochberg false discovery rate (FDR) method was used for multiple comparison corrections in both tests, and FDR-threshold *q* < 0.05 was chosen for statistical significance (Benjamini and Hochberg, 1995).

## Results

**FIGURE 1:**
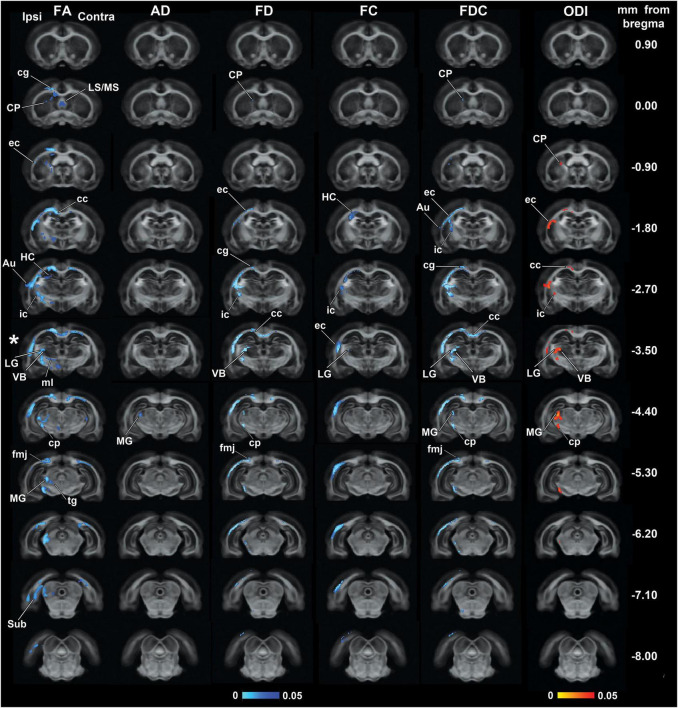
Whole-brain group differences in FA, AD, FD, FC, FDC, and ODI metrics when comparing sham-operated and mTBI rats after 35 days of the sham-operation or injury. Areas displaying significant group differences in mTBI versus sham-operated rats (TFCE, *p*-value < 0.05, FWE-corrected) are overlaid on the FOD-based template. Significances are displayed as voxels in a light-dark blue scale, representing mTBI group values lower than sham-operated group values, and in a red-yellow scale, representing mTBI group values higher than sham-operated group values. The asterisk shows the epicenter of the lesion. Au, auditory cortex; cc, corpus callosum; cg, cingulum; cp, cerebral peduncle; CP, caudate putamen; ec, external capsule; fmj, forceps minor of the corpus callosum; HC, hippocampus; ic, internal capsule; LG, lateral geniculate nuclei; LS/MS, lateral/medial septal nucleus; MG, medial geniculate nucleus; ml, medial lemniscus; Sub, subiculum; tg, tegmental nuclei; VB, ventrobasal complex.

Figure 1 shows the outcomes from the group analyses in FA, AD, FD, FC, FDC, and ODI maps when comparing mTBI and sham-operated rats. The epicenter of the primary lesion in mTBI animals was at approximately −3.50 mm from bregma (asterisk in Figure 1), where we found the most extensive significant changes between the groups. The secondary damage expanded into connected areas throughout the brain at 35 days after the initial injury. These five parameters showed significant decreases (TFCE, *p*-value < 0.05, FWE-corrected) in mTBI rats compared to sham-operated ones. From the DTI parameters, FA significantly decreased in both white and gray matter areas throughout the brain and AD showed few highlighted voxels in the midbrain. FD and FDC showed similar pattern of changes than FA, while the results in FC were restricted to few damaged areas. ODI significantly increased in the closed areas to the primary injury, mainly white matter and thalamic areas, which were also shown in FA, FD, and FDC maps.

**FIGURE 2:**
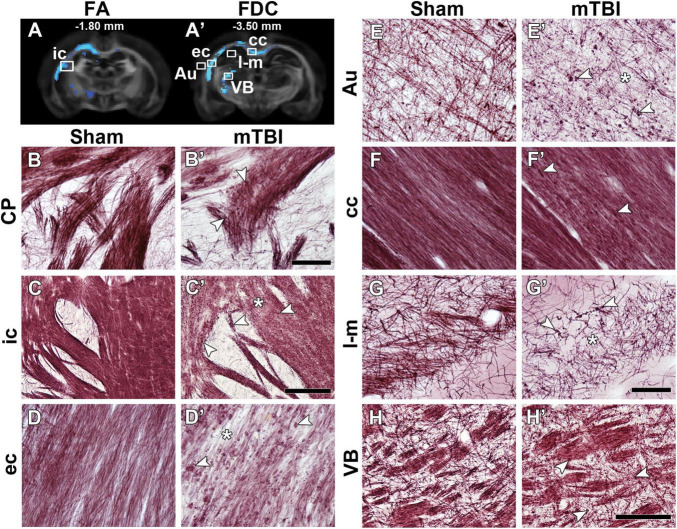
FA **(A)** and FDC **(A’)** at –1.80 and –3.50 mm from bregma, respectively, from the group analyses. White squares indicate the location of the photomicrographs shown in **(B–H)**. Representative photomicrographs of myelin-stained sections of a sham-operated and a mTBI rat from the caudate putamen **(B,B’)** from –0.90 mm (not shown in **A**), internal capsule **(C,C’)** from –1.80 mm, and external capsule **(D,D’)**, auditory cortex **(E,E’)**, corpus callosum **(F,F’)**, stratum-lacunosum moleculare **(G,G’)**, and ventrobasal complex **(H,H’)** from –3.50 mm from bregma. White arrowheads point at myelin alterations associated with axonal damage and asterisks indicate areas with extensive decrease in density of myelinated axons. AI values of these two animals shown in this figure are shown in Supplementary Table 1. Au, auditory cortex; cc, corpus callosum; CP, caudate putamen; ec, external capsule; ic, internal capsule; l-m, stratum-lacunosum moleculare; VB, ventrobasal complex. Scale bars: 50 μm **(B,B’,D,D’–G,G’)**, 150 μm **(H,H’)**, and 250 μm **(C,C’)**.

Figure 2 shows representative photomicrographs of myelin-stained sections from a sham-operated and mTBI rat 35 days after the sham-operation or injury. We observed two microstructural alterations in mTBI animals when compared to sham-operated rats: axonal alterations shown as dark accumulation of staining (arrowheads in Figure 2) and decreased axonal density (asterisks in Figure 2). Axonal alterations are associated to axonal injury and/or myelin damage, which were consistently found in all areas exhibiting group differences, in all the animals. We observed wide-spread axonal alterations rostrally in the brain, such as in the caudate putamen (arrowheads in Figure 2B’), which appeared more numerous and closer to the epicenter of the primary lesion, as in the internal capsule (Figure 2C’), external capsule (Figure 2D’), cortex (Figure 2E’), and ventrobasal complex (Figure 2H’). Decrease in axonal density was found in the internal capsule (Figure 2C’), external capsule (Figure 2D’), auditory cortex (Figure 2E’), and stratum-lacunosum moleculare (Figure 2G’). In the corpus callosum, we observed axonal alterations along the structure ipsi- and contralaterally, and a decrease in axonal density (Figure 2F’); however, not so prominent as in other areas. We also observed the thinning of fiber bundles in the internal capsule at the level of the caudate putamen (arrow in Figure 2C’).

**FIGURE 3:**
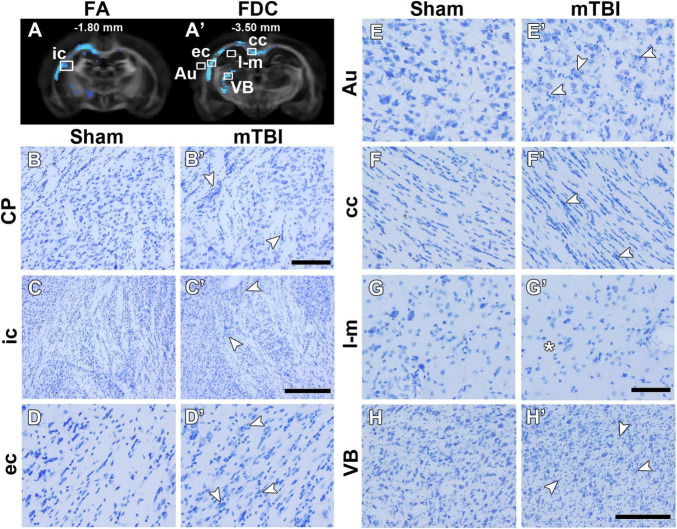
FA **(A)** and FDC **(A’)** at –1.80 and –3.50 mm from bregma, respectively, from the group analyses. White squares indicate the location of the photomicrographs shown in **(B–H)**. Representative photomicrographs of Nissl-stained sections of a sham-operated and a mTBI rat from the caudate putamen **(B,B’)** from –0.90 mm (not shown in **A**), internal capsule **(C,C’)** from –1.80 mm, and external capsule **(D,D’)**, auditory cortex **(E,E’)**, corpus callosum **(F,F’)**, stratum-lacunosum moleculare **(G,G’)**, and ventrobasal complex **(H,H’)** from –3.50 mm from bregma. White arrowheads point at gliosis alterations associated with axonal damage and asterisk indicates less cell density. CD values of these two animals shown in this figure are shown in Supplementary Table 1. Au, auditory cortex; cc, corpus callosum; CP, caudate putamen; ec, external capsule; ic, internal capsule; l-m, stratum-lacunosum moleculare; VB, ventrobasal complex. Scale bars: 50 μm **(B,B’,D,D’–G,G’)**, 150 μm **(H,H’)**, and 250 μm **(C,C’)**.

Figure 3 shows representative photomicrographs of Nissl-stained sections from a sham-operated and mTBI rat 35 days after the sham-operation or injury. We found a wide-spread increase in cell density, associated to gliosis, rostrally in the brain as in the caudate putamen (Figure 3B’). Gliosis was more evident closer to the epicenter of the primary lesion, as in the internal capsule (Figure 3C’), external capsule (Figure 3D’), cortex (Figure 3E’), corpus callosum (Figure 3F’), and ventrobasal complex (Figure 3H’). We observed a decrease in cell density in the stratum-lacunosum moleculare (Figure 3G’) along with the loss of fiber bundles in the layer (asterisk in Figure 2G’).

**FIGURE 4:**
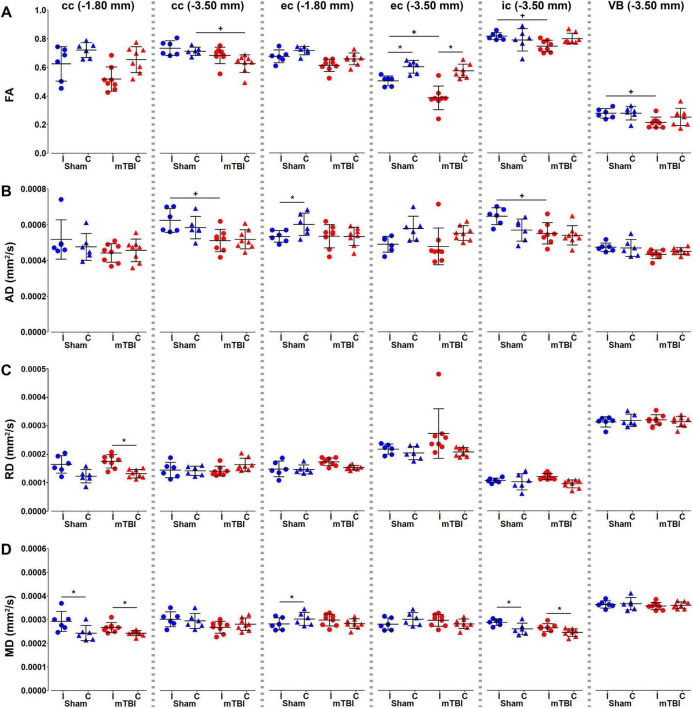
Diffusion tensor imaging (DTI) metrics, fractional anisotropy **(A)**, and axial **(B)**, radial **(C)**, and mean **(D)** diffusivities, were analyzed in the corpus callosum and external capsule at –1.80 and –3.50 mm, and the internal capsule and ventrobasal complex at –3.50 mm from bregma. Sham-operated animals are indicated in blue and mTBI in red, while ipsi- and contralateral hemispheres are represented by circles and triangles, respectively. Results are shown as mean and standard deviation, and paired (*) *t*-test comparing ipsi- and contralateral sides within animals (*q < 0.05) or unpaired (+) *t*-test comparing the same hemisphere between sham-operated and mTBI rats (^+^*q* < 0.05), both FDR-corrected. AD, axial diffusivity; cc, corpus callosum; ec, external capsule; FA, fractional anisotropy; MD, mean diffusivity; RD, radial diffusivity; ic, internal capsule; VB, ventrobasal complex.

**FIGURE 5:**
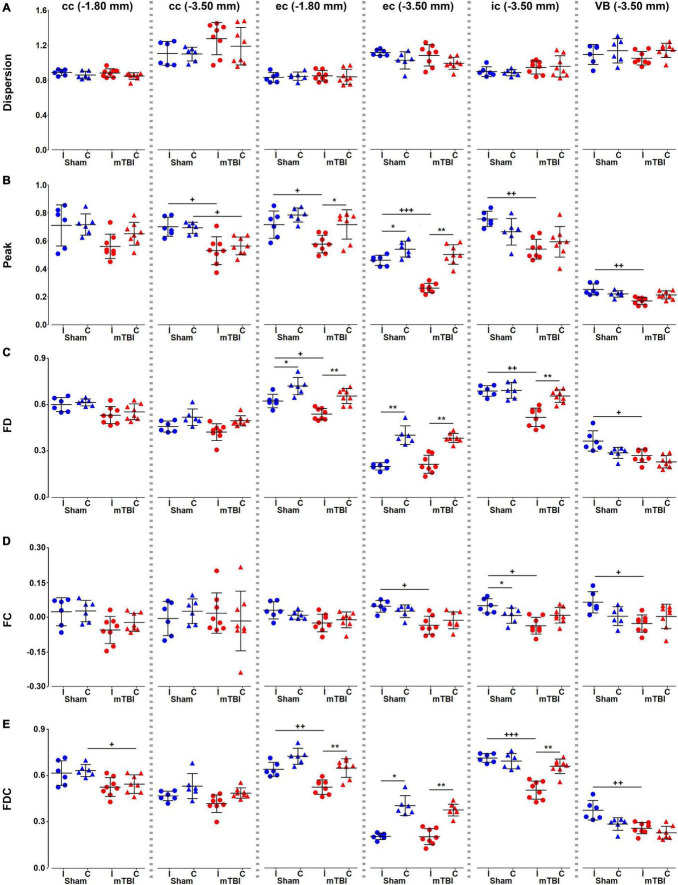
Fixel-based metrics, dispersion **(A)**, peak FOD amplitudes **(B)**, FD **(C)**, FC **(D)**, and FDC **(E)**, were analyzed in the corpus callosum and external capsule at –1.80 and –3.50 mm, and the internal capsule and ventrobasal complex at –3.50 mm from bregma. Sham-operated animals are indicated in blue and mTBI in red, while ipsi- and contralateral hemispheres are represented by circles and triangles, respectively. Results are shown as mean and standard deviation, and paired (*) *t*-test comparing ipsi- and contralateral sides within animals (*q < 0.05, **q < 0.01) or unpaired (+) *t*-test comparing the same hemisphere between sham-operated and mTBI rats (^+^*q* < 0.05, ^++^*q* < 0.01, ^+++^*q* < 0.001), both FDR-corrected. cc, corpus callosum; ec, external capsule; FC, fiber bundle cross-section; FD, fiber density; FDC, fiber density and fiber bundle cross-section; ic, internal capsule; VB, ventrobasal complex.

**FIGURE 6:**
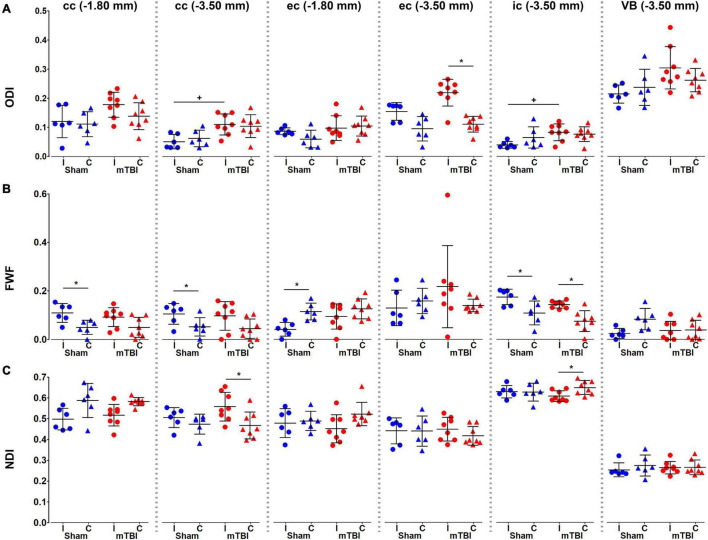
NODDI metrics, ODI **(A)**, FWF **(B)**, and NDI **(C)**, were analyzed in the corpus callosum and external capsule at –1.80 and –3.50 mm, and the internal capsule and ventrobasal complex at –3.50 mm from bregma. Sham-operated animals are indicated in blue and mTBI in red, while ipsi- and contralateral hemispheres are represented by circles and triangles, respectively. Results are shown as mean and standard deviation, and paired (*) *t*-test comparing ipsi- and contralateral sides within animals (*q < 0.05) or unpaired (+) *t*-test comparing the same hemisphere between sham-operated and mTBI rats (^+^*q* < 0.05), both FDR-corrected. cc, corpus callosum;ec, external capsule; ic, internal capsule; FWF, free water fraction; NDI, neurite density index; ODI, orientation dispersion index; VB, ventrobasal complex.

We performed an ROI analysis of specific brain regions on MRI maps at the perilesional and epicenter sites; −1.80 and −3.50 mm from bregma, respectively (Figures 4–6). We found significant decrease in FA ipsilaterally in mTBI rats when comparing within mTBI animals and/or between sham-operated and mTBI rats at −3.50 mm from bregma (Figure 4A). AD decreased ipsilaterally in the mTBI rats in the corpus callosum and internal capsule at the epicenter (Figure 4B). On the contrary, RD (Figure 4C) and MD (Figure 4D) increased at −1.80 mm in the corpus callosum and/or at −3.50 mm in the internal capsule. It is worth noting that sham-operated rats ipsilaterally showed significant differences as compared to the contralateral side in the ROI analysis, such as FA and AD in the external capsule (Figures 4A,B), MD in the corpus callosum and external capsule at −1.80 mm, or internal capsule (Figure 4D).

We did not find significant differences in dispersion values in any of the brain areas (Figure 5A). Peak FOD amplitudes showed a significant decrease ipsi- and/or contralaterally, when comparing sham-operated and mTBI rats or hemispheres within mTBI rats (Figure 5B). FD, FC, and FDC showed decreased values ipsilaterally in almost all the brain areas in mTBI rats; specially in those at the level of the epicenter of the primary lesion at −3.50 mm (Figures 5C–E). Also, a decrease was obtained ipsilaterally in the sham-operated animals, such as in FD and FDC in the external capsule (Figures 5C,E) or in FC in the internal capsule (Figure 5D).

We found significant increases in ODI values in the corpus callosum, external capsule and internal capsule at −3.50 mm (Figure 6A). FWF showed a significant increase in the internal capsule, when comparing when comparing ipsi- and contralateral hemispheres in mTBI rats, and also in the sham-operated rats in the corpus callosum, external and internal capsule (Figure 6B). NDI showed an increase in the corpus callosum at −3.50 mm, and a decrease in the internal capsule when comparing ipsi- and contralateral hemispheres in mTBI rats (Figure 6C).

**FIGURE 7:**
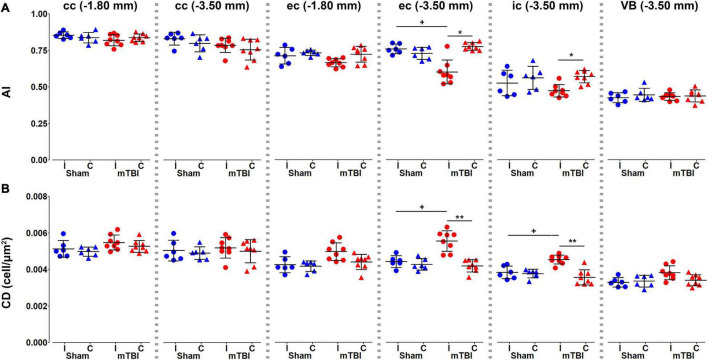
Histology metrics, anisotropy index **(A)** and cellular density **(B)**, were analyzed in the corpus callosum and external capsule at –1.80 and –3.50 mm, and the internal capsule and ventrobasal complex at –3.50 mm from bregma. Sham-operated animals are indicated in blue and mTBI in red, while ipsi- and contralateral hemispheres are represented by circles and triangles, respectively. Results are shown as mean and standard deviation, and paired (*) *t*-test comparing ipsi- and contralateral sides within animals (*q < 0.05, **q < 0.01) or unpaired (+) *t*-test comparing the same hemisphere between sham-operated and mTBI rats (^+^*q* < 0.05), both FDR-corrected. AI, anisotropy index; cc, corpus callosum; CD, cellular density; ec, external capsule; ic, internal capsule; VB, ventrobasal complex.

Histological analysis showed that anisotropy index significantly decreased in the external and internal capsule at −3.50 mm (Figure 7A), where we found increased cellularity (Figure 7B).

Table 1 shows the correlation results when comparing MRI and histology metrics from the ROI analyses. In the corpus callosum at −3.50 mm, AI showed a negative correlation with RD (*R* = −0.544; *q* = 0.014) (Supplementary Figure 1A). In the external capsule, we found that cellularity correlated positively with RD (*R* = 0.474; *q* = 0.044) at −1.80 mm from bregma (Supplementary Figure 1B). At −3.50 mm, the external capsule showed the most robust correlations. AI positively correlated with FA (*R* = 0.737; *q* = 9.623 × 10^–5^), peak FOD amplitude (*R* = 0.781; *q* = 1.558 × 10^–5^), FD (*R* = 0.473; *q* = 0.044), and FDC (*R* = 0.492; *q* = 0.034), and negatively with ODI (*R* = −0.753; *q* = 4.780 × 10^–5^) (Supplementary Figures 1C,E,G,I,K). Also, cell density correlated negatively with FA (*R* = −0.712; *q* = 2.235 × 10^–4^), peak FOD amplitude (*R* = −0.695; *q* = 3.656 × 10^–4^), FD (*R* = −0.491; *q* = 0.034) and FDC (*R* = −0.505; *q* = 0.027), and positively with ODI (*R* = 0.709; *q* = 2.100 × 10^–4^) (Supplementary Figures 1D,F,H,J,L). The internal capsule showed correlations between CD and FD (*R* = −0.571; *q* = 0.008), and FDC (*R* = −0.580; *q* = 0.007) (Supplementary Figures 1M,N). The ventrobasal complex showed a negative correlation between CD and FC (*R* = −0.467; *q* = 0.048) (Supplementary Figure 1O).

**TABLE 1:** Coefficient (R) and *q*-values (q) from the Pearson’s correlations between dMRI and histological metrics.

		cc (−1.80 mm)		cc (−3.50 mm)		ec (−1.80 mm)		ec (−3.50 mm)		ic (−3.50 mm)		VB (−3.50 mm)	
		AI	CD	AI	CD	AI	CD	AI	CD	AI	CD	AI	CD
**FA**	***R***	0.086	−0.061	0.429	−0.016	0.237	−0.333	**0.737**	−**0.712**	0.013	−0.344	0.045	−0.333
	***q***	0.809	0.877	0.075	0.961	0.401	0.187	**9.623 × 10** ^–^ ^5^ ***	**2.235 × 10** ^–^ ^4^ ***	0.962	0.172	0.915	0.187
**AD**	***R***	0.156	0.166	0.018	−0.165	0.026	0.051	0.436	−0.357	−0.033	−0.098	0.350	−0.348
	***q***	0.643	0.616	0.961	0.621	0.951	0.898	0.070	0.154	0.926	0.775	0.164	0.166
**RD**	***R***	−0.017	0.150	−**0.544**	−0.040	−0.228	**0.474**	−0.410	0.434	−0.076	0.347	0.188	0.119
	***q***	0.961	0.660	0.014*	0.924	0.426	0.044*	0.093	0.071	0.835	0.168	0.554	0.738
**MD**	***R***	0.199	0.258	−0.251	−0.167	−0.121	0.282	−0.040	0.086	−0.099	0.057	0.360	−0.057
	***q***	0.523	0.350	0.367	0.616	0.735	0.287	0.924	0.809	0.775	0.882	0.149	0.882
**Disp**	***R***	0.039	0.153	−0.248	−0.058	−0.082	−0.172	−0.175	0.049	−0.055	−0.115	0.459	−0.026
	***q***	0.926	0.651	0.373	0.882	0.818	0.603	0.591	0.903	0.887	0.744	0.053	0.951
**Peak**	***R***	0.151	0.079	0.329	0.036	0.231	−0.181	**0.781**	−**0.695**	0.072	−0.189	0.193	−0.383
	***q***	0.657	0.829	0.193	0.926	0.417	0.573	**1.558 × 10** ^–^ ^5^ ***	**3.656 × 10** ^–^ ^4^ ***	0.846	0.551	0.540	0.123
**FD**	***R***	0.084	−0.089	0.105	−0.122	0.257	−0.423	**0.473**	−**0.491**	0.443	−**0.571**	0.038	−0.302
	***q***	0.813	0.805	0.768	0.734	0.354	0.081	0.044*	0.034*	0.066	0.008**	0.926	0.248
**FC**	***R***	0.102	−0.277	−0.125	−0.461	0.149	−0.147	0.324	−0.261	0.125	−0.416	0.103	−**0.467**
	***q***	0.770	0.299	0.725	0.051	0.660	0.660	0.201	0.342	0.725	0.087	0.769	0.048*
**FDC**	***R***	0.093	−0.103	0.177	−0.139	0.266	−0.413	**0.492**	−**0.505**	0.419	−**0.580**	0.018	−0.374
	***q***	0.792	0.769	0.588	0.687	0.330	0.090	0.034*	0.027*	0.084	0.007**	0.961	0.133
**ODI**	***R***	−0.167	−0.001	−0.089	0.190	−3.290 × 10^–4^	−0.042	−**0.753**	**0.709**	0.028	0.200	−0.150	0.434
	***q***	0.598	0.997	0.791	0.537	0.999	0.912	**4.780 × 10** ^–^ ^5^ ***	**2.100 × 10** ^–^ ^4^ ***	0.947	0.515	0.633	0.071
**FWF**	***R***	0.355	0.416	0.161	0.190	0.103	0.026	^–^0.321	0.371	−0.202	0.209	0.313	0.148
	***q***	0.160	0.088	0.611	0.537	0.761	0.948	0.215	0.138	0.511	0.490	0.230	0.635
**NDI**	***R***	0.002	−0.078	0.435	0.301	0.218	−0.421	−0.230	0.292	0.030	−0.308	−0.164	0.095
	***q***	0.995	0.818	0.071	0.255	0.462	0.083	0.425	0.272	0.941	0.243	0.603	0.775

Numbers in bold indicate the correlations significantly different from zero: *q < 0.05; **q < 0.01; ***q < 0.001. AD, axial diffusivity; AI, anisotropy index; cc, corpus callosum; CD, cellular density; Disp, dispersion; ec, external capsule; FA, fractional anisotropy; FC, fiber bundle cross-section; FD, fiber density; FDC, fiber density and fiber bundle cross-section; FWF, free water fraction; ic, internal capsule; MD, mean diffusivity; NDI, neurite density index; ODI, orientation dispersion index; RD, radial diffusivity; VB, ventrobasal complex.

## Discussion

In this study, we used DTI, FBA, and NODDI to investigate microstructural changes in the mildly injured brain *ex vivo*. Whole-brain analyses revealed significant differences throughout the rat brain when comparing sham-operated and mTBI brains. FA, FD, and FDC showed a similar pattern of microstructural changes in the brain in the voxel-based analysis; however, FBA metrics showed more significant fixels in white matter, FA also detected changes in gray matter, while ODI mainly detected areas closest to the primary lesion. Myelin- and Nissl-stained sections of the same brains revealed axonal alterations and increased cellularity in white and gray matter areas. Quantitative histological analyses of selected brain areas revealed that alterations in myelinated axons and increased cellularity correlated to changes in MRI metrics after mTBI.

Our study demonstrates that DTI provides a good sensitivity to detect microstructural-associated changes associated to mTBI in both white and gray matter (Bennett et al., 2012; Hylin et al., 2013; Stemper et al., 2015; Herrera et al., 2017; Hutchinson et al., 2018). Previous studies demonstrated the presence of axonal damage and increased cellularity due to gliosis using histology analysis (Mac Donald et al., 2007a,b; Hylin et al., 2013; San Martín Molina et al., 2020). More specifically, decreased FA and AD due to axonal damage, and increased RD have been associated to gliosis. In our study, the positive association observed between FA, AD and AI in the corpus callosum and external capsule were indicative of axonal damage and/or axonal loss, while the negative association observed between FA and CD in the external capsule was associated to axonal damage in conjunction with a marked increase in cellularity. This was further indicated by the inverse relationship between RD and AI, and the positive association between RD and CD in both the corpus callosum and external capsule. With regards to CSD-derived metrics, the positive association observed in the external capsule between peak FOD amplitudes, FD and FD, and AI, and their negative relationship with CD, could be attributed a reduction in intact healthy fiber bundles and gliosis in those areas. Interestingly, the positive relationship observed between dispersion and AI in the ventrobasal complex was contrary to that observed in the white matter. As the ventrobasal complex is a region comprising of multiple fiber bundle populations; we hypothesize that the situation could result either from a reduction in densities of the primary fiber bundle or an increase in the densities of the secondary fibers (Riffert et al., 2014; Grazioplene et al., 2018). The NODDI analysis provided information of the microstructural compartment contributions, which has shown promising results in clinical research (Kamiya et al., 2020). Our voxel-based analysis only showed statistical differences in ODI in areas showing decreased FA, as suggested in previous studies (Jespersen et al., 2012; Zhang et al., 2012). Based on histology, we expected to also obtain differences in both NDI and FWF; however, it has been described that the reproducibility of NODDI in rat brain at 9.4 T was lower for FWF than ODI and NDI, which might require a considerably larger number of animals to observe biological changes in those parameters after mild TBI (McCunn et al., 2021).

Previous studies in animal models have shown the potential of DTI to detect the effect of mild injury in the brain. Wright et al. (2017) showed that FBA detected more voxels in the rat brain after severe injury than DTI *in vivo*. These authors found reduced FD predominantly in the ipsilateral corticospinal tract, external capsule, fimbria, and corpus callosum, which also extended along the corpus callosum from the ipsilateral to contralateral hemisphere after severe TBI in rats. Additionally, these authors performed track-weighted imaging measurements revealing significantly fewer streamlines, shorter and straighter trajectories in the ipsilateral corpus callosum, fimbria, and internal capsule in TBI rats as compared to sham-operated ones. In accordance with our study, fixel-based metrics showed more significant voxels in the white matter as compared to FA. However, FA showed more significant voxels in the gray matter as compared to fixel-based metrics. Our results indicate that fixel- and DTI-based analyses may offer complementary information of tissue changes after mTBI as already shown in a previous study on adolescents with moderate-to-severe TBI (Verhelst et al., 2019). Our study also showed that ODI mainly detected changes in areas closed to the primary lesion. NODDI has demonstrated to be sensitive to early acute microstructural changes following a single closed head controlled cortical impact in rats, not detectable by DTI (McCunn et al., 2021). Another study using a closed-skull impact in mice showed greater sensitivity using NODDI than DTI to microstructural changes in white matter associated to astrocyte and microglia (Gazdzinski et al., 2020). Human studies suggested that the combination of DTI and NODDI significantly enhances our understanding of white matter microstructural alterations in subacute and chronic TBI (Muller et al., 2021; Oehr et al., 2021). In summary, our study suggests that the combination of dMRI parameters may provide more complete information of changes in tissue microstructure after brain injury, and therefore, an evaluation including different approaches could provide better understanding of the tissue damage after brain injury.

Different sensitivity of DTI- and fixel-based analyses could potentially result from poor fixel correspondence in complex brain areas, such as crossing fibers or gray matter, thereby resulting in large intra-group variances. Conventional DTI studies are typically based on lower diffusion weightings resulting in decreased angular contrast (Soares et al., 2013). The FBA framework, which is conditionally valid in the high *b*-value regime (≥3,000 s/mm^2^) (Raffelt et al., 2012; Genc et al., 2020), provides an improved resolution of crossing fiber bundle populations (Tournier et al., 2013). As, the diffusion-weighted signal originating from a restricted compartment is nearly fully preserved (Hall and Alexander, 2006; Yeh et al., 2010), and high *b*-values attenuate the signal from the extracellular water, the diffusion-weighted signal which is assumed to be restricted in the radial direction (Stanisz et al., 1997; Assaf and Basser, 2005; Alexander, 2008; Assaf et al., 2008; Barazany et al., 2009; Alexander et al., 2010) corresponds to the intra-axonal water-content (Raffelt et al., 2012). Moreover, as the FOD amplitude is relatively equal to the total radial diffusion-weighted signal, it provides an approximate measure of the intra-axonal volume of the corresponding fiber bundle (Raffelt et al., 2012). Therefore, the estimated FD metric is largely derived from the anisotropic WM component of the diffusion MRI signal and, is thereby highly specific to changes in axonal density (Tournier et al., 2004, 2007; Raffelt et al., 2012). Moreover, we also demonstrated the use of CSD-derived metrics for providing a better understanding of the tissue-specific sources underlying the microstructural alterations post mTBI. Our findings were consistent with previous literature, as changes in conventional DTI-based metrics can be influenced by several pathological events, not just decreased density, such as axonal injury, gliosis, edema, or increased membrane permeability (Mac Donald et al., 2007a; Budde et al., 2011; Bennett et al., 2012; Salo et al., 2017). Future studies may benefit by the incorporation of signal fractions representative of tissue-specific microstructure (Khan et al., 2020; Mito et al., 2020). It is worth mentioning that advanced methodologies come at the cost of more complex data processing; however, these methods can provide more specific information on tissue microstructure and pathological alterations in the context of brain diseases, disorders, and injuries, and specially with a more comprehensive histological validation.

We used higher *b*-value than typically used *in vivo*, as it has been shown that diffusivity values *ex vivo* are decreased 2-3-fold as compared to *in vivo* due to changes in (1) temperature and (2) tissue microstructural properties following chemical fixation (Sun et al., 2003; Rane and Duong, 2011; Wu et al., 2013; Wang et al., 2018). As opposed to *in vivo* conditions, wherein the extra-axonal water signal attenuates with increasing *b*-value, the presence of immobile water confined in bodies of glial cells and other minute compartments such as vesicles, has been postulated in the extra-axonal space *ex vivo* (Stanisz et al., 1997; Veraart et al., 2019). Therefore, despite studies using high *b*-values enable a more direct comparison with *in vivo* results, further *in vivo* studies of mTBI using advanced dMRI are needed to provide more insights into the potential of these imaging methods (Wright et al., 2017; San Martín Molina et al., 2020; Pham et al., 2021).

While we have used CFE to correct for multiple comparisons, CFE applies on each measure separately that may give rise to false positives over the nominal alpha-threshold due to testing several parameters per a fixel or a voxel (Smith et al., 2021). However, given the complexity of CFE correction, it is challenging to decide what is the best approach to strike balance between Type I and Type II errors in the analysis. As a mitigation approach in this work, we used histology to verify the findings of DTI, FBA, and NODDI analyses. We also note that the statistically significant differences in the maps were in locations where we would expect to see differences based on previous experiments on how the tissue damage expands from the cortical primary lesion to connected areas as secondary damage. We used FWE correction when performing (potentially highly correlated) voxel- and fixel-based analyses as there exist a potentially large number of false positives that arise with data-informed clustering, such as TFCE. However, in the ROI-based analysis, where the ROIs were defined based on the brain anatomy, we used FDR that is more liberal than FWE. Our reason for using FDR with ROIs is that it controls the number of false positives effectively, while simultaneously having a considerably lower number of false negatives than FWE. This balancing act, in turn, may lead to better reproducibility than strict control for false positives as argued by Geerligs and Maris (2021).

Our study included animals in the subacute stage, 35 days after the injury, which were subset of our previous *in vivo* study (San Martín Molina et al., 2020). This subgroup of animals showed, at day 3, hyperintensity associated to edema in the somatosensory cortex on *in vivo* T2-weighted images in four out of eight mTBI rats and hypointensity related to parenchymal bleeding in one of these four rats. After 28 days, three out of eight rats showed persistent but less pronounced cortical edema as compared to the acute time point, even two out of those three rats still showed signs of bleeding. The complex cortical patterns at the level of the primary injury associated to the secondary injury, and more importantly, to pathological outcomes warrant future studies. Despite of cortical damage in T2-weighted MRI, all the animals presented a consistent pattern of secondary tissue damage throughout the brain based on histology. Although the dura of the sham-operated and mTBI animals remained intact and no brain tissue damage was inflicted during the craniotomy, differences between ipsi- and contralateral hemispheres in sham-operated animals were reported. Histologically, the sham-operated rats did not present any tissue damage or cellular alteration. However, the sham-operation involving craniotomy might cause effects, e.g., swelling, that could affect the measurements at acute and/or subacute stages. Future studies should consider including a group of naïve rats to discern between minor sham-operation alterations and tissue damage associated to the injury.

Human histopathological studies are very scarce, and the low mortality after mTBI does not allow histopathological examination to correlate with MRI experiments in mTBI patients. Preclinical experiments including histology provide an opportunity to understand the mTBI pathogenesis (Bennett et al., 2012; Hylin et al., 2013; Stemper et al., 2015; Haber et al., 2017; Yu et al., 2017; San Martín Molina et al., 2020; Sinke et al., 2021). In our study, there were axonal alterations observed in all the areas highlighted in dMRI analyses, which were consistent across all the mTBI animals. These findings demonstrate that significant differences between sham-operated and mTBI rats in dMRI analyses reflected ongoing microstructural alterations after injury.

## Conclusion

This study reveals the potential of the dMRI framework to detect microstructural alterations when comparing sham-operated and rats post mTBI. The combination of dMRI-based analyses could provide a more complete insight into the detection of microstructural alterations in white and gray matter after mild injuries. Advanced methodologies in combination with histopathological characterization of the same subjects can open new avenues into the improvement of our understanding of dMRI, which can directly enhance the diagnosis and prognosis of the mildly injured brain.

## Data Availability Statement

The raw data supporting the conclusions of this article will be made available by the authors, without undue reservation.

## Ethics Statement

The animal study was reviewed and approved by the Animal Ethics Committee of the Provincial Government of Southern Finland and in accordance with the guidelines of the European Community Council Directives 86/609/EREC.

## Author Contributions

KC, OG, and AS contributed to study concept and design. KC, ON, RS, IS, JT, and MA contributed to data acquisition and analyses. All authors contributed to interpretation of the data, drafting, and reviewing the manuscript, and provided final approval for publication.

## Conflict of Interest

The authors declare that the research was conducted in the absence of any commercial or financial relationships that could be construed as a potential conflict of interest.

## Publisher’s Note

All claims expressed in this article are solely those of the authors and do not necessarily represent those of their affiliated organizations, or those of the publisher, the editors and the reviewers. Any product that may be evaluated in this article, or claim that may be made by its manufacturer, is not guaranteed or endorsed by the publisher.

## Footnotes

1
http://nitrc.org/projects/noddi_tolbox

## Supplementary Material

Supplementary Figure 1 Pearson’s correlations between dMRI and histological metrics statistically significant in Table 1, for the corpus callosum **(A)**, external capsule **(B–L)**, internal capsule **(M,N)** and ventrobasal complex **(O)**. Sham-operated animals are indicated in blue and mTBI in red, while ipsi- and contralateral hemispheres are represented by circles and triangles, respectively. AI, anisotropy index; cc, corpus callosum; ec, external capsule; FA, fractional anisotropy; FD, fiber density; FDC, fiber density and fiber bundle cross-section; ic, internal capsule; ODI, orientation dispersion index; RD, radial diffusivity; VB, ventrobasal complex.

## Abbreviations

AD: axial diffusivity
AI: anisotropy index
CD: cellular density
CFE: connectivity-based fixel enhancement
CSD: constrained spherical deconvolution
CT: computed tomography
DTI: diffusion tensor imaging
dMRI: diffusion magnetic resonance imaging
DW-GRASE: diffusion-weighted gradient- and spin-echo
FA: fractional anisotropy
FBA: fixel-based analysis
FC: fiber bundle cross-section
FD: fiber density
FDC: fiber density and fiber bundle cross-section
FOD: fiber orientation distribution
FWE: family-wise error
FWF: free water fraction
GM: gray matter
HARDI: high-angular-resolution diffusion imaging
LFP: lateral fluid percussion
MD: mean diffusivity
MRI: magnetic resonance imaging
mTBI: mild traumatic brain injury
NODDI: neurite orientation dispersion and density imaging
NDI: neurite density index
ODI: orientation dispersion index
PBS: phosphate-buffered saline
RD: radial diffusivity
ROI: region-of-interest
ST: structure tensor
TFCE: threshold-free cluster enhancement
VBA: voxel-based analyses
WM: white matter.
