# Supplementary figures and images for: Microstructural Tissue Changes in a Rat Model of Mild Traumatic Brain Injury
Source: Front Neurosci. 2021 Nov 26;15:746214. doi: 10.3389/fnins.2021.746214 (PMC8662623; doi:10.3389/fnins.2021.746214)

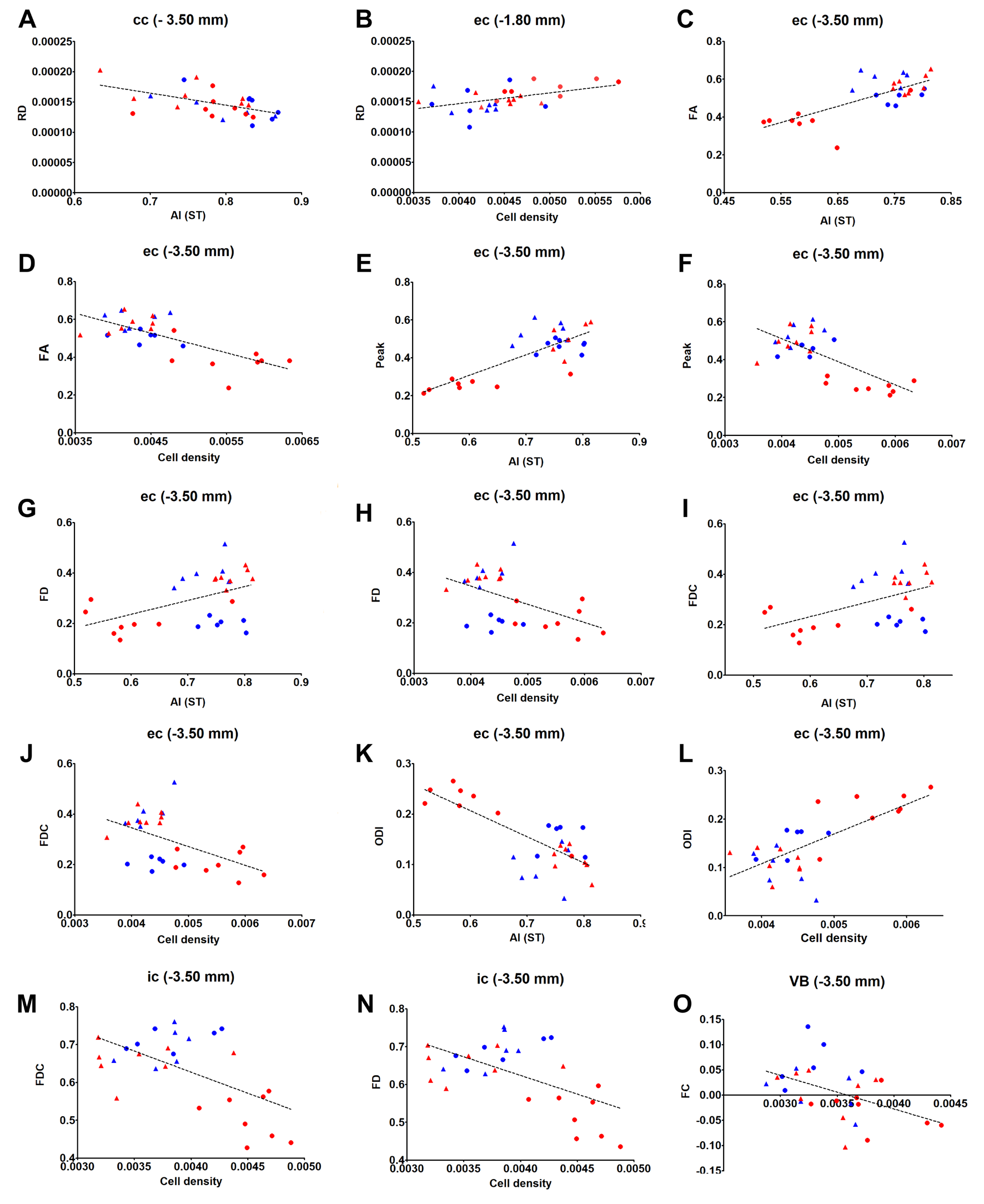

Supplement: Supplementary Figure 1 — Pearson’s correlations between dMRI and histological metrics statistically significant in Table 1, for the corpus callosum (A), external capsule (B–L), internal capsule (M,N) and ventrobasal complex (O). Sham-operated animals are indicated in blue and mTBI in red, while ipsi- and contralateral hemispheres are represented by circles and triangles, respectively. AI, anisotropy index; cc, corpus callosum; ec, external capsule; FA, fractional anisotropy; FD, fiber density; FDC, fiber density and fiber bundle cross-section; ic, internal capsule; ODI, orientation dispersion index; RD, radial diffusivity; VB, ventrobasal complex. [file Image_1.TIF]
