# Supplementary material for: Microstructural Tissue Changes in a Rat Model of Mild Traumatic Brain Injury
Source: Front Neurosci. 2021 Nov 26;15:746214. doi: 10.3389/fnins.2021.746214 (PMC8662623; doi:10.3389/fnins.2021.746214)
Supplement: Supplementary file 2 [file Table_1.DOCX]

**Supplementary Table S1:** Anisotropy index (AI) and cellular density (CD) values obtained from the histological analyses from the ipsilateral hemisphere of the sham-operated and mTBI animals shown in Figure 2 and 3, respectively.

|  | **Animal** | **AI** | **CD** |
| --- | --- | --- | --- |
| **cc (-1.80 mm)** | **Sham** | 0.856 | 0.00597 |
|  | **mTBI** | 0.814 | 0.00556 |
| **cc (-3.50 mm)** | **Sham** | 0.745 | 0.00544 |
|  | **mTBI** | 0.783 | 0.00546 |
| **ec (-1.80 mm)** | **Sham** | 0.722 | 0.00409 |
|  | **mTBI** | 0.621 | 0.00450 |
| **ec (-3.50 mm)** | **Sham** | 0.752 | 0.00492 |
|  | **mTBI** | 0.649 | 0.00553 |
| **ic (-3.50 mm)** | **Sham** | 0.641 | 0.00384 |
|  | **mTBI** | 0.425 | 0.00448 |
| **VB (-3.50 mm)** | **Sham** | 0.456 | 0.00301 |
|  | **mTBI** | 0.444 | 0.00429 |
